# Mastering Death: The Roles of Viral Bcl-2 in dsDNA Viruses

**DOI:** 10.3390/v16060879

**Published:** 2024-05-30

**Authors:** Chathura D. Suraweera, Benjamin Espinoza, Mark G. Hinds, Marc Kvansakul

**Affiliations:** 1 Genome Sciences and Cancer Division, The John Curtin School of Medical Research, Australian National University, Canberra 2601, Australia; chathura.suraweera@anu.edu.au; 2 Department of Biochemistry and Chemistry, La Trobe University, Melbourne, VIC 3086, Australia; 17992336@students.latrobe.edu.au; 3 Bio21 Molecular Science and Biotechnology Institute, The University of Melbourne, Parkville, VIC 3052, Australia

**Keywords:** apoptosis, autophagy, Bcl-2, virus, innate immunity, structure

## Abstract

Proteins of the Bcl-2 family regulate cellular fate via multiple mechanisms including apoptosis, autophagy, senescence, metabolism, inflammation, redox homeostasis, and calcium flux. There are several regulated cell death (RCD) pathways, including apoptosis and autophagy, that use distinct molecular mechanisms to elicit the death response. However, the same proteins/genes may be deployed in multiple biochemical pathways. In apoptosis, Bcl-2 proteins control the integrity of the mitochondrial outer membrane (MOM) by regulating the formation of pores in the MOM and apoptotic cell death. A number of prosurvival genes populate the genomes of viruses including those of the pro-survival Bcl-2 family. Viral Bcl-2 proteins are sequence and structural homologs of their cellular counterparts and interact with cellular proteins in apoptotic and autophagic pathways, potentially allowing them to modulate these pathways and determine cellular fate.

## 1. Introduction

Regulated cell death (RCD) is utilized by the metazoan cell to control its fate and ensure the removal of damaged or unwanted cells from the organism. Multiple RCD mechanisms exist and include necroptosis, pyroptosis, and macroautophagy, and, while the genetic and mechanistic bases differentiating these RCD pathways continue to be described, it is clear that the complex nature of RCD involves tight regulation and cross-talk between the various RCD types to enable correct function [1,2,3]. Viral manipulation of RCD occurs through two processes in particular, apoptosis and macroautophagy, and both pathways play important roles in modulating cell fate during viral infection and replication. Macroautophagy, more generally referred to as autophagy, is a major catabolic pathway to remove unwanted cellular components by their encapsulation in autophagosomes and transport to lysosomes, where they undergo enzymatic degradation [1]. While autophagy is a cell survival mechanism, its inhibition accelerates RCD [1]. Apoptosis, on the other hand, is a form of RCD that functions through the activation of a cascade of cysteine aspartyl proteases (caspases) that disassemble key components of the cell and lead to its eventual phagocytosis [1]. In combination, autophagy and apoptosis form part of the innate immunity response to pathogen invasion [4,5], and both pathways invoke the B-cell lymphoma-2 (Bcl-2) family of genes. Viruses have adapted to modulate apoptosis and autophagy in response to infection and their genomes frequently contain Bcl-2 genes.

Apoptosis is critical in metazoans for maintaining healthy cell populations by silently clearing unwanted cells following development or destroying damaged cells [6,7,8,9]. Dysregulation of apoptosis has been shown to be important in many disorders including cancer and autoimmune and developmental diseases, as well as being a fundamental aspect of viral infection and replication [10,11,12,13,14,15]. Apoptosis is regulated via two main pathways: the extrinsic or death receptor-mediated pathway [16] and the intrinsic or mitochondrially mediated pathway [8,9]. The latter pathway is controlled by the interplay of Bcl-2 proteins, and both pathways converge at the level of caspases, where a hierarchical protease cascade commencing with initiator caspases is initiated that triggers the activation of executioner caspases, which ultimately dismantle the cell. Extrinsic apoptosis has been recently reviewed in [16] and, here, we focus on the role of viral Bcl-2 proteins in intrinsic apoptosis and their potential role in autophagy. 

**Figure 1:**
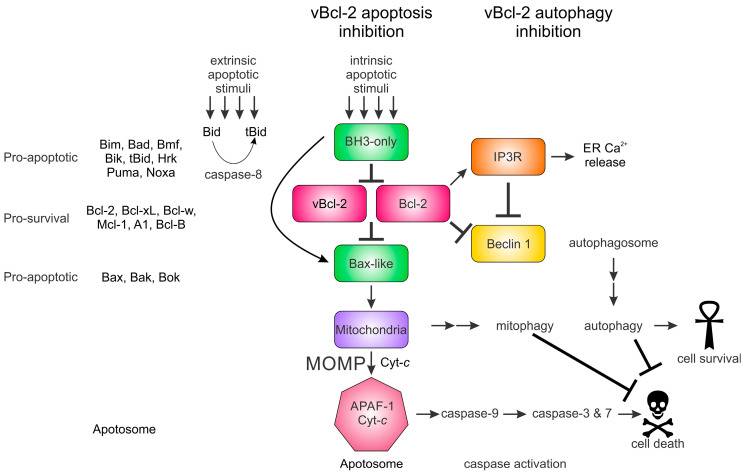
Bcl-2 family proteins and their involvement in intrinsic apoptosis and potential in autophagy signaling. The Bcl-2 family proteins (classified and named on the left) are functionally differentiated by their protein sequence into pro-apoptotic or pro-survival members through the presence of Bcl-2 homology (BH) motifs 1 through 4. The BH3-only protein Bid potentially links extrinsic apoptosis via extracellular signals through its proteolytic activation by caspase 8 to form the BH3-only protein truncated Bid (tBid). Multiple apoptotic stimuli lead to the upregulation of the pro-apoptotic BH3-only and Bax-like proteins that lead to mitochondrial outer membrane permeabilization (MOMP). The release of Cytochrome *c* initiates caspase activation via the formation of the apoptosome, which, ultimately, leads to cell death. Viral Bcl-2 and cellular Bcl-2 family proteins can bind and neutralize the pro-apoptotic proteins and inhibit apoptosis. A second, less well-understood mechanism of survival is for the pro-survival proteins to inhibit autophagosome through interaction with Beclin 1 and prevent autophagosome assembly and thus autophagic-induced cell death. Bcl-2 proteins have also been associated with the inositol 1, 4, 5 triphosphate receptor, a major regulator of cellular calcium flux that also plays a role in autophagy.

**Figure 2:**
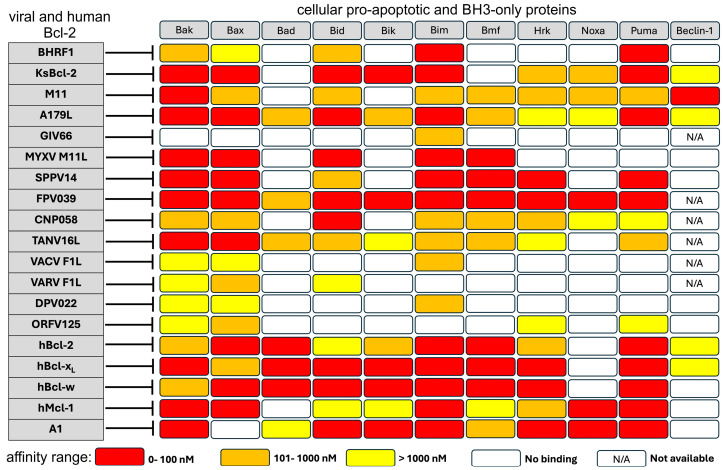
Comparison of BH3 motif peptide-binding profiles of viral Bcl-2 proteins with cellular Bcl-2 homologs. The binding profiles of vBcl-2 proteins included EBV BHRF1 [46,47], KSHV KsBcl-2 [46,48], murine gammaherpes virus M11 [49], ASFV A179L [39], GIV66 [50], MYXV M11L [51], sheeppox virus SPPV14 [52], Fowlpox virus FPV039 [53], Canarypox virus CNP058 [54], tanapox virus TANV16L [55], VACV F1L [56], VARV F1L [57], deerpox virus DPV022 [58], and Orf virus ORFV125 [59]. Cellular Bcl-2 proteins included Bcl-2, Bcl-x_L_, Bcl-w, Mcl-1 [60,61], and A1 [62]. The sequences of all BH3 motif peptides used were of human origin, except for GIV66, where the BH3 motif peptides were of zebrafish origin. FPV039 and CNP058 used BH3 motif peptides of avian origin. The color scheme indicates the binding affinity ranges from 0 to 100 nM in red, 101 to 1000 nM in orange, and >1000 nM in yellow; white indicates no binding and N/A indicates data not available, as shown in the inset below the table. Pro-survival viral and mammalian Bcl-2 proteins are named on the left and their pro-apoptotic binding partners are named across the top of the figure.

**Figure 3:**
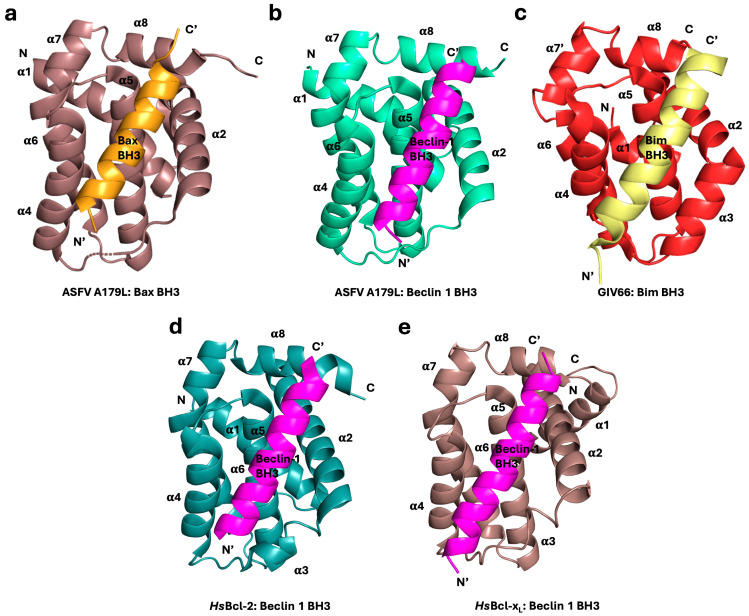
Structural comparison of viral Bcl-2:pro-apoptotic BH3 peptide complexes as well as Bcl-2:Beclin 1 BH3 peptide complexes and their human counterparts Bcl-2 and Bcl-x_L_. Viral Bcl-2 proteins bind BH3 ligands using the same binding groove, and many of the intermolecular interactions are conserved. Cartoon diagrams of complexes of Bcl-2 proteins (**a**) ASFV A179L (dark salmon) in complex with Bax BH3 peptide (orange) (PDB ID 5UA5), (**b**) ASFV A179L (green) in complex with Beclin 1 BH3 peptide (magenta) (PDB ID 6TZC) [63], (**c**) grouper iridovirus GIV66 (red) in complex with Bim BH3 peptide (pale yellow) (PDB ID 5VMO), (**d**) human Bcl-2 (dark green) in complex with Beclin 1 BH3 peptide (magenta) (PDB ID 5VAX), and (**e**) human Bcl-x_L_ (dark salmon) in complex with Beclin BH3 peptide (magenta) (PDB ID 2PON) [64]. All views in (**b**–**e**) are oriented as in (**a**). Helices are labeled α1–α8. The view is into the hydrophobic binding groove formed by helices α2–α5. Images were generated using the PYMOL Molecular Graphics System, Version 1.8 Schrodinger, LLC.

The Bcl-2 family arose early in metazoan history and this highly conserved protein family is present from the basal metazoans (phylum Porifera, the sponges) through to higher metazoans [7]. The mechanism of action of the Bcl-2 family also appears to be conserved from the earliest point in metazoan evolution. Three-dimensional structures have been solved for BH3-complexes and Bcl-2 proteins from porifera [17], trichoplax [18], and cnidaria (Hydra) [7], as well as from mice and humans [19,20], revealing a high degree of conservation between ancient and modern metazoans. The Bcl-2 family is central to mitochondrial-mediated apoptosis (Figure 1) and characterized by the presence of 1–4 highly conserved short-sequence regions, termed Bcl-2 homology (BH) motifs, that differentiate the Bcl-2 family members into pro-survival and pro-apoptotic activities [1,9,21]. Pro-apoptotic Bcl-2 are further subdivided into those Bcl-2 members that feature multiple BH motifs, termed the multi-motif executioners or Bax-like proteins, and those that feature only the BH3 motif, which are referred to as BH3-only proteins (Figure 1). Mechanistically, the interplay of Bcl-2 proteins is dominated by the interactions of the BH3 motif with a hydrophobic binding groove on multi-motif Bcl-2 proteins and many interactions have been quantitated (Figure 2). The BH3 motif is an amphipathic sequence that harbors four conserved hydrophobic residues (*h*1–*h*4), with *h*2 almost invariably represented by a leucine residue [21,22]. This BH3 motif binds as a helical element in a groove provided by the pro-survival protein (Figure 3). Differences in the *h*1, *h*3, and *h*4 residues therefore appear to provide affinity and selectivity differences between Bcl-2 members [23]. Interactions occur between binding with the canonical hydrophobic groove of Bcl-2 proteins, comprised of eight α-helices with a hydrophobic surface region formed from α2–α5, with the α5 helix at its center [13,24] (Figure 3). 

Following cellular stress such as infection, BH3-only proteins (Bim, Bik, Bad, Bid, Bmf, Noxa, Hrk, and Puma) are activated and inhibit the multi-motif pro-survival Bcl-2 proteins (Bcl-2, Bcl-x_L_, Bcl-w, Mcl-1, A1, and Bcl-B), which function as inhibitors of the pro-apoptotic members (Bax, Bak, and Bok) (Figure 1) [21,22]. Many Bcl-2 proteins carry a hydrophobic C-terminal transmembrane domain that allows for their localization to the mitochondrial outer membrane (MOM), where Bax and Bak oligomerize to form macromolecular pore complexes leading to MOM permeabilization (MOMP) [25] and the release of Cytochrome *c* and other apoptogenic factors from the mitochondria [8,9]. This step is considered the point of no return for the cell, and MOMP ultimately leads to the activation of the caspase cascade (Figure 1) [26,27,28]. Some Bcl-2 proteins such as Bok play less direct roles in apoptosis and are associated with other cellular processes. Bok is localized to the endoplasmic reticulum (ER) and strongly associated with the inositol 1, 4, 5 triphosphate receptor (IP3R) [29] (Kd 65 nM, [30]), a key determinant in cellular calcium regulation that is itself also closely associated with Beclin 1, a component required for autophagosome formation [31]. In addition, Bok is associated with Mcl-1 in an interaction occurring through their C-terminal transmembrane regions [32]. Thus, the Bcl-2 family is not only associated with apoptosis through direct interaction with other Bcl-2 proteins but also with cellular calcium flux and Beclin 1-regulated autophagosome formation (discussed below) through the IP3R receptor system. These findings show the potential complexity of the Bcl-2-fold protein regulation of key cellular processes.

Autophagy most likely arose in the last eukaryote common ancestor [33], placing it much earlier in evolution than apoptosis [7]. Accumulating evidence suggests that the Bcl-2 proteins, in addition to their key role in intrinsic apoptosis regulation, also play other less well-understood roles in autophagy, cellular senescence, metabolism and redox, and Ca^2+^ homeostasis. Autophagy, like apoptosis, forms part of the innate immune response [4]. Among the components of the autophagy pathway is Beclin 1, a key component in the formation of autophagosomes. Beclin 1 bears a BH3-like motif, engages Bcl-2 proteins, and is required for autophagosome assembly (reviewed in [34]). Beclin 1 antagonism by Bcl-2 proteins prevents the formation of a multi-protein complex necessary for autophagosome assembly and autophagy initiation. While the importance of the cellular Bcl-2:Beclin 1 interaction has been disputed (discussed in [34]), nevertheless, studies on viruses suggest a role of viral Bcl-2 proteins in regulating autophagy activation.

It is now well established that many double-stranded DNA (dsDNA) viruses, such as those from *Asfarviridae*, *Iridoviridae*, *Herpesvirale*, and *Poxviridae*, employ sophisticated mechanisms to avoid the cellular innate immune response. Approximately half the genomes of poxviruses such as the smallpox (variola) virus (VARV) are postulated to be involved in modulating host–virus interactions [35]. Included among these innate immunity-modulating viral genes are those that mimic Bcl-2 (vBcl-2; Figure 1), which subverts or promotes apoptosis, thereby allowing the virus to exploit host cell machinery for replication [13,15]. There is also accumulating evidence that viral Bcl-2 (vBcl-2) proteins inhibit autophagy, a process that can also induce a form of RCD [1]. While Bcl-2 proteins have a key role to play in regulating apoptosis, they play a less well-defined role in regulating autophagy. The apoptosis–autophagy crosstalk has its focus on mitochondria and the association between Bcl-2 family members and Beclin 1. Viruses, through their acquisition of Bcl-2 genes, have developed an ability to regulate these mechanisms. Here, we discuss the roles viral Bcl-2 proteins might play and potential molecular mechanisms of action.

## 2. Viral Bcl-2 Genes and the Regulation of Apoptosis

The regulation of cellular apoptosis is critical to many aspects of viral infection, and assimilated viral Bcl-2 genes frequently mimic the activity of pro-survival cellular Bcl-2 genes while avoiding the regulation directed at the cellular proteins. 

### 2.1. Asfarviridae

The only member of the family *Asfarviridae*, African Swine Fever Virus (ASFV) is a large double-stranded (ds) DNA virus capable of expressing over 150 proteins [36,37], with a genome in the range of 170–194 kbp. ASFV infection leads to a fatal hemorrhagic fever in domestic pigs with a near 100% mortality rate, though it is benign in the natural sylvatic cycle between African warthogs and *Ornithodoros* ticks, its vector [38]. ASFV is known to produce numerous immunomodulatory proteins, many of which remain uncharacterized; however, a series of recent studies have shed light on the potential immune regulatory effects and established roles of some of the known ASFV virulence factors [39,40,41].

The ASFV genome encodes a single Bcl-2 homolog, A179L, a 21 kDa protein that binds a range of cellular Bcl-2 proteins (Figure 2) and co-localizes with porcine tBid within the perinuclear space and cytoplasm [42]. The structure of A179L is homologous to mammalian Bcl-2 proteins (Figure 3), and BH3 binding similarly occurs in a surface groove, with many of the intermolecular interactions conserved between the viral and mammalian Bcl-2 proteins (Figure 3a,b,d,e). Expression of A179L in either human (HeLa, Myeloid Leukemia) or monkey (BSC-40, Vero) cells [42,43,44] showed that A179L expression was capable of inhibiting apoptosis in these heterologous systems. Biochemical studies showed that peptides based on BH3 motif sequences from all pro-apoptotic Bcl-2 proteins of boar (*Sus scrofa*) bind A179L in the nanomolar to micromolar affinity range [39]. These results indicated that A179L has the potential to inhibit most cellular apoptotic pathways, and the interactions have been subsequently examined using live virus experiments in porcine primary macrophages [41].

Reis et al. (2023) found that deletion of the A179L gene from the virulent Benin 97/1 (BeninΔA179L) strain of ASFV impaired viral replication and resulted in the earlier onset of apoptosis in vitro when compared to the parent strain. Analyses of biomarkers for apoptosis induction including the upregulation of expression of apoptotic effector caspases 3 and 7, DNA fragmentation, and cell surface marker annexin V were consistent with the increased induction of apoptosis in BeninΔA179L-infected cells compared to the wild-type virus [41]. A surprising observation in the study was the apparent increase in the uptake of BeninΔA179L by macrophages in vitro over the virulent strain, which the authors suggest may be mediated by an interaction between viral envelope-incorporated phosphatidylserine and host macrophage cell surface receptors [41]. Pigs challenged with BeninΔA179L showed promising results, with a reduction in viral replication of the attenuated strain and subclinical symptoms; yet, despite this, immunization with repeated booster applications of BeninΔA179L failed to provide protection to pigs when subsequently challenged with the parental strain [41]. 

Shi et al. (2021), analyzed the levels of apoptotic cells compared to necroptotic (a form of death receptor-mediated RCD [1]) cells in vitro following infection with the DNA virus HSV-1 and two RNA viruses, Influenza A and Sendai virus, in the presence of A179L [45]. Cells were transfected with A179L, and it was observed that in addition to the established role of A179L in inhibiting apoptosis following infection with all three viruses, A179L-expressing cells challenged with a viral infection also exhibited significantly higher levels of necroptosis compared to mock-transfected cells [45]. While the inhibition of apoptosis facilitated viral replication in the early stages of infection, in late stages, virus release occurred through plasma membrane permeabilization induced by necroptosis. In a recombinant vaccinia virus, A179L is expressed at early and late times post-infection [43], consistent with this finding. Though many molecular details are absent, these results suggest that A179L has the potential to regulate multiple cell death pathways.

### 2.2. Iridoviridae

The *Iridoviridae* are divided into α-iridoviruses whose primary vectors are arthropods and aquatic animals such as amphibians and fish, while the β-iridoviruses primarily infect insects and crustaceans. Economically, α-iridoviruses are particularly significant, with aquaculture enterprises at particular risk, with numerous viruses known to infect fish stocks. Several members of the *Iridoviridae* encode modulators of intrinsic apoptosis [65]. For example, the tiger frog virus (TFV), an a-iridovirus of the genus ranavirus, contains an open reading frame (ORF), ORF145R, a putative vBcl-2, though, unusually, this protein only contains an identifiable BH1 motif and transmembrane region [66]. Despite this, sequence analysis shows some level of identity with other iridovirus species encoding vBcl-2 as well as the pro-survival Bcl-2 proteins present in the African clawed frog, *Xenopus laevis* [66]. He et al. (2019) postulate that the mitochondrial localization of TFV vBcl-2 suggests a role in the direct inhibition of intrinsic apoptosis [66]. Chinese giant salamander (*Andrias davidianus*) iridovirus (GSIV) has been shown to regulate apoptosis in vitro, with GSIV promoting both intrinsic and extrinsic pathways of apoptosis in infected cells [67], with further in vitro studies by Li et al. (2021) showing that the Chinese giant salamander Bcl-x_L_ was capable of limiting GSIV infection when overexpressed in cultured giant salamander muscle cells [68].

The grouper iridovirus (GIV) vBcl-2, GIV66, has previously been shown to inhibit intrinsic apoptosis by downregulating host pro-death Bcl-2 proteins, localized primarily at the mitochondrial membrane of the host cell and exerting early control over apoptosis during infection [69,70]. Structural data and interaction studies revealed that GIV66, in contrast to most Bcl-2 proteins, is highly selective by binding only to proapoptotic Bim [50] (Figure 2), though the same BH3-binding mechanism is used as that of other Bcl-2 family members (Figure 3c). Unusually, GIV66 forms a homodimer utilizing the canonical BH3 ligand binding groove, a previously unobserved phenomenon in Bcl-2 family proteins, which then dissociated upon binding to Bim, resulting in a 1:1 GIV66:Bim heterodimer [50]. This raises the question of whether such groove-to-groove homodimerization of GIV66 impedes the viral inhibition of apoptosis in vivo; clarification of this will require further investigation [50].

### 2.3. Adenoviridae

The *Adenoviridae* family has been a pivotal group of viruses in aiding our understanding of DNA viruses’ ability to modulate cell death. Human adenovirus was first isolated in 1953 from human adenoid tissue [71]. Then, 30 years after the discovery of the virus, the earliest evidence of a viral Bcl-2 from the gene *e1b* was described, a 19 kDa protein, E1B19K, which inhibited apoptosis. Functional studies initially observed the localization of E1B19K to the nuclear membrane of infected cells [72], while later studies demonstrated the ability of this vBcl-2 to disrupt apoptosis through the direct inhibition of Bax and Bak at the MOM [73,74,75]. Though one of the first viral Bcl-2 proteins described, to date, there have been no structural studies on this protein describing the exact molecular interactions with its ligands.

### 2.4. Poxviridae

The *Poxviridae* are a family of large dsDNA viruses that replicate exclusively in the cytoplasm and therefore encode all the genes necessary for DNA replication and transcription [76]. Poxviridae are divided into *Entomopoxvirinae*, which infect insects, and *Chordopoxvirinae*, which infect invertebrates and diverse vertebrates, including humans, respectively [77]. Amongst the members of the chordopoxvirus family are viruses such as smallpox (variola) and monkeypox, which present a significant threat to humans. Currently, there are 17 genera forming the chordopoxvirus subfamily. The genomes of poxviruses are ~128–360 kbp or approximately 320 open reading frames in size and represent a proteome of 120–300 proteins [78]. Approximately half of the genome of poxviruses is dedicated to accessory genes that encode immunomodulatory proteins, many of which are important for host immune evasion [35,77,79]. Four paralogous accessory gene families, which include TNF-receptor domains, Kelch repeat proteins, ANK proteins, and Bcl-2 proteins, have been identified in shaping viral modulation of the host immune response [35,77,79].

**Figure 4:**
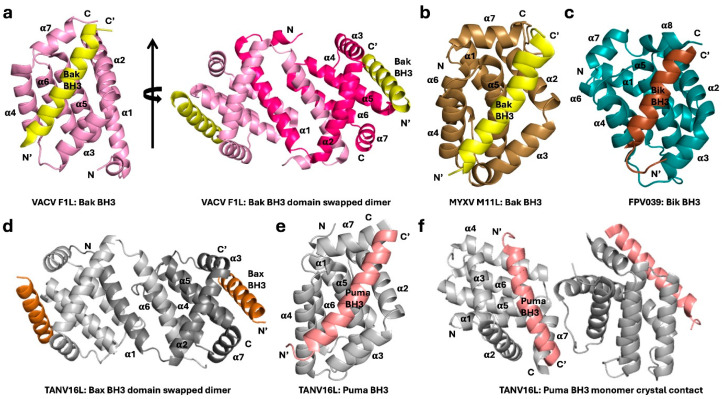
Poxvirus-encoded Bcl-2 proteins are protomerically divergent and may exist as monomers or domain-swapped dimers. Cartoon representation of a comparison of different poxvirus Bcl-2 proteins. Although the protomer structure varies, the BH3 ligand binds in the BH3 binding groove provided by the Bcl-2 homolog. (**a**) Structure of vaccinia virus (VACV) F1L (pink) in complex with Bak BH3 peptide (yellow) (PDB ID 4D2L) [80]. VACV F1L helices are labeled α1–α7. The view on the left in (**a**) is of the hydrophobic binding groove formed by helices α2–α5 of a protomer. On the right, the view is down the 2-fold symmetry axis between the domain-swapped α1 helices of the dimer. The second protomer is colored dark pink. The vertical arrow represents the axis of rotation and the broad arrow the rotation about the axis. (**b**) Myxoma virus (MYXV) M11L (sand) in complex with the Bak BH3 peptide (yellow) (PDB ID 2JBY) [51]. (**c**) Fowl poxvirus FPV039 (teal) in complex with the Bik BH3 peptide (brown) (PDB ID 5TZP) [53]. (**d**) Tanapoxvirus TANV16L (grey) domain-swapped dimer in complex with the Bax BH3 peptide (dark orange) (PDB ID 6TRR). (**e**) TANV16L (grey) monomer in complex with the Puma BH3 peptide (salmon) (PDB ID 6TQP). (**f**) TANV16L (grey) in complex with the Puma BH3 peptide (salmon) (PDB ID 6TQP), showing the crystal packing contacts of the neighboring protomer [55].

Bcl-2 proteins that play a crucial role in regulating host cell apoptosis and modulate programmed cell death and genes encoding for putative Bcl-2 proteins are found in the genomes of many poxviruses that mimic the structure and/or function of their pro-survival cellular counterparts [15,77]. Like other viral Bcl-2 proteins, the poxviral Bcl-2 family is highly homologous with mammalian Bcl-2 proteins and interaction with their targets occurs via a surface groove (Figure 4). Despite the conserved function of viral Bcl-2 proteins, these proteins are often highly divergent in their sequences, making it difficult to identify them by examining their sequences alone. However, experimental structural determination of many of these putative viral Bcl-2 homologs revealed that they are highly structurally conserved and mimic both the structure and function of cellular Bcl-2 proteins (Figure 4) [15,77].

Vaccinia virus (VACV), the prototypical member of *Poxviridae*, encodes multiple proteins that are known or hypothesized to possess structures resembling the Bcl-2-fold [77], though not all such proteins have a defined role in apoptosis. VACV F1L is an anti-apoptotic vBcl-2 protein, as shown by its ability to inhibit staurosporin-induced apoptosis [81]. Furthermore, a viral deletion mutant lacking the *f1l* gene, referred to as VV811, experienced apoptosis, whereas the expression of F1L effectively halted all downstream events from apoptosis after the mitochondria [81,82]. The same study also showed that VACV F1L tethered to the mitochondrial outer membrane through its C-terminal hydrophobic transmembrane (TM) domain [81]. In vitro experiments of VACV F1L revealed that it has a very restricted subset of interactions with pro-apoptotic Bcl-2 members. F1L binds Bax, Bak, and Bim BH3 motif peptides with sub-micromolar affinities [56] (Figure 2) and potentially reflects the exact apoptotic pathways inhibited by this protein. Primarily, F1L inhibits apoptosis via the recruitment of Bim in a live viral infection [80]. Additionally, F1L has been shown to act as a functional ortholog of cellular Mcl-1 [83] by preventing Bax and Bak homo-oligomerization at the MOM and the downstream activation of cell death [82]. Structural studies of F1L demonstrate that it features a conserved Bcl-2 fold consisting of seven α-helices and the α2-α5 helices form the hydrophobic canonical ligand-binding groove, which provides the interaction site for the pro-apoptotic BH3 motif peptide (Figure 4a) [56,80]. However, F1L forms a dimer in solution with an unusual domain-swapped topology, where the α1 helix of one protomer is swapped with the α1 helix of a neighboring protomer to make the dimer interface [56]. In addition to its ability to dimerize, F1L utilizes a unique N-terminal extension (residues 1–56), which is reported to be required for caspase-9 inhibitory activity [84] and was predicted to be helical [85]. However, subsequent structural studies revealed that the extended N-terminal region of F1L is intrinsically disordered and did not engage in apoptosis regulation in cells [86]. Site-directed mutagenesis of the F1L binding groove residue A115W (at the end of the α3 helix) completely blocked the interaction with Bim_L_ BH3, but not that with Bak BH3, and was unable to stop apoptosis [80].

Gerlic et al. showed that the N-terminal region of VACV F1L directly interacts with inflammasome regulator NLRP1 and inhibits inflammasome activation in vitro [87]. These data were further supported by an in vivo functional assay using infected macrophages with an F1L knockout (KO) virus that promoted caspase-1 activation and IL-1β secretion compared to its wild-type virus [87]. These results suggest that F1L utilizes an alternative pathway to inhibit apoptosis to promote virulence [87]. The F1L deletion mutant of Modified Vaccinia Ankara (MVA) virus, MVA-ΔF1L, was shown to have a protective immunization role against a mouse Ectromelia virus (ECTV)—a mousepox virus—infection model, and the immunization of mice with MVA-ΔF1L prevented death from a lethal mousepox infection. This suggests that an F1L deletion virus could be a potential vaccine candidate against poxvirus infection [88]. More recent work from Pelin et al. demonstrated that the F1L KO virus showed increased immunogenic apoptosis and increased VACV safety by initiating the secretion of interleukin (IL)-1β cytokines in a variety of glioblastoma cells as well as multiple human cancer cell lines (NCI-60) [89], thus promoting virus-induced apoptosis. In combination, these data suggest that the F1L KO virus could be used as an oncolytic agent for cancer therapy [89].

A second Bcl-2 homolog in vaccinia virus, VACV N1L, is a 117 residue Bcl-2-like apoptosis inhibitory protein that lacks any significant sequence identity with VACV F1L or its cellular counterparts [90,91]. N1L lacks a C-terminal TM domain, a feature of many Bcl-2 proteins that target the MOM, and its absence therefore localizes N1L to the cytosol rather than the MOM [90]. N1L inhibits apoptosis through high-affinity interactions with pro-apoptotic proteins Bim, Bid, and Bak similar to those reported for the cellular pro-survival protein Bcl-x_L_ [90,91]. Additionally, N1L inhibits NF-κB signaling, important in the immune response during infection [92,93], and this appears to be the primary function of N1L [92]. However, a structural study of N1L revealed that these two apoptotic events are mediated in two independent sites on N1L [92,93]. These data were further supported by the N1L co-IP experiment with cellular Bax, Bad, and Bid, where Bax was expressed by cellular transfection [91]. N1L adopted an overall Bcl-2-fold structure and was homodimeric in solution. The crystal structure revealed seven α helices in each protomer. In contrast to the F1L domain-swapped dimer, the N1L dimer interface was formed by the interaction between the α1 and α6 helices [90].

Variola virus (VARV) F1L is a homolog of VACV F1L (88% shared identity). However, notwithstanding the high shared sequence identity, their mode of apoptosis inhibition is different. VARV F1L primarily inhibits apoptosis through interaction with Bak, Bax, and Bid, but does not interact with Bim [57] (Figure 2). Functional data demonstrated that VARV F1L could only inhibit host intrinsic apoptosis via sequestering Bax but not with Bak-mediated apoptosis [57]. These data demonstrate the complexity of predicting the activity of Bcl-2 members based on sequence similarity alone.

The Bcl-2 protein EMV025 is encoded by Ectromelia virus, the causative agent of mousepox, and is an F1L homolog that interacts with Bax, Bak, and Bim and inhibits Bak-mediated apoptosis during mousepox infection [94]. Although the exact molecular mechanisms are to be determined, apoptosis induced by Ectromelia virus infection is partially regulated by autophagy [95,96]. The inhibition of Beclin 1-induced autophagy results in caspase-independent cell death, showing that if apoptosis is inhibited, then autophagy potentially contributes to cell death [96].

Myxoma virus (MYXV), a prototypical member of the *Leporipoxviridae* family [97], is a causative agent of myxomatosis, a lethal disseminated disease primarily infecting European rabbits [98]. MYXV encodes a vBcl-2 homolog, M11L, which lacks detectable sequence similarity with cellular Bcl-2 and is localized at the MOM via the C-terminal hydrophobic TM domain (Figure 4b). M11L has the remarkable ability to interact with multiple subsets of host pro-apoptotic Bcl-2 proteins, including Bax, Bak, Bim, and Bid [51,99] (Figure 2), and a functional study has demonstrated that M11L primarily operates by sequestering Bak and Bax [51]. This result stands in contrast to the mechanism of action of vaccinia virus F1L, which primarily acts through Bim sequestration [80]. The crystal structure of M11L revealed a unique compact monomeric Bcl-2 fold with seven α-helices with a conserved ligand-binding groove to engage pro-apoptotic Bcl-2 proteins (Figure 4b) [51,99]. Furthermore, the expression of M11L in virus-infected HEK293 cells has been shown to block the Fas-ligand-induced extrinsic apoptosis and downstream activation of the caspase cascade [100]. These data suggest that M11L not only targets host mitochondrial-mediated apoptosis but also extrinsic apoptosis.

The *Captipoxviridae* are responsible for diseases such as lumpy skin disease, sheeppox, goatpox, and deerpox and pose a significant economic burden in domesticated ruminants, particularly in developing countries [101]. The most widely studied member of this virus family is the sheeppox virus, a virus that encodes SPPV14, a vBcl-2 homolog that appropriates host intrinsic apoptosis [52]. SPPV14 lacks any significant shared sequence identity with its cellular counterparts and in vitro interaction studies showed that it interacts with almost all cellular pro-apoptotic Bcl-2 proteins with nanomolar to sub-micromolar affinities except Noxa [52,102] (Figure 2). Structural studies revealed that SPPV14 exists as a monomeric Bcl-2-fold protein similar to M11L [102]. Functional studies showed that the expression of SPPV14 could inhibit apoptosis induced by diverse stimuli such as UV irradiation, etoposide, FasL, or retroviral infection in Phoenix Ecotropic packaging cells and Cytochrome *c* release from Jurkat cells [52]. Deerpox virus-encoded DPV022 is another anti-apoptotic Bcl-2 gene that lacks recognizable BH motifs. Unlike the sheeppox homolog SPPV14, DPV022 binds a highly restricted subset of pro-apoptotic Bcl-2 proteins: Bak, Bax, and Bim [103] (Figure 2), a binding range similar to that previously observed for VACV F1L [56]. The crystal structure of DPV022 features a domain-swapped dimeric configuration with an overall conserved Bcl-2 fold, a feature previously observed in VACV F1L and VARV F1L [56,57,58]. The *Captiviridae* Bcl-2 proteins, like their orthopox homologs, have a range of binding repertoires in addition to dimeric structural motifs.

ORF virus is the prototypical member of the *Parapoxviridae*, a family that commonly infects sheep, goats, and humans, causing unique skin infections [104]. The ORF virus genome encodes viral Bcl-2 homolog ORFV125 [105], which, while inhibiting apoptosis and caspase activation [106], lacks obvious sequence features of Bcl-2 proteins such as BH motifs. ORFV125 features a C-terminal hydrophobic motif, which is sufficient to localize it to the MOM, and is essential for anti-apoptotic function. Immunoprecipitation experiments showed that ORFV125 interacted with a selective subset of pro-apoptotic proteins, Bax, Bim, Puma, Hrk, Bik, and Noxa, but not with Bak [107]. In vitro affinity experiments of ORFV125 reported that it selectively interacts with BH3 motif peptides of Bak, Bax, Puma, and Hrk with sub-micromolar affinities (Figure 2). Interestingly, no interaction was observed with Bim, a near-universal binder of mammalian pro-survival Bcl-2 proteins [108]. Like some other poxviral Bcl-2 proteins, ORFV125 exists in a domain-swapped dimeric configuration [59,108], with an overall conserved Bcl-2 fold and dimer topology, as previously seen in VACV F1L, VARV F1L, and DPV022 [56,57,58].

Two members of the avian-infecting *Avipoxviridae* genus were shown to encode a putative Bcl-2 homolog, avipoxviruses canarypox virus (CNPV) CNP058, and fowlpox virus (FPV) FPV039, which can readily be identified by their conserved BH1 motifs [53]. Structural studies of both proteins have shown that they adopt a conserved monomeric Bcl-2 fold with seven alpha helices (Figure 4c) [53,54], as previously seen for M11L [51] and SPPV14 [102]. A functional study of CNP058 showed that the expression of CNP058 could inhibit radiation-induced (UV) apoptosis in HeLa cells [54]. Similarly, FPV039 was shown to inhibit apoptosis when it was overexpressed with all other BH3-only proteins [109]. Furthermore, it was demonstrated that FPV039 interaction with Bax prevents Bax oligomerization and subsequent cell death [109]. In contrast, in vitro interaction studies of CNP058 showed that it interacts with a distinct subset of pro-apoptotic Bcl-2 proteins and did not show significant interactions with Bad Bik or Bok [54] (Figure 2). These results provide the possible mechanism of action of apoptosis inhibition by both FPV039 and CNP058 as proceeding via the sequestration of Bim and direct interaction with cellular Bax and Bak [53,54].

Tanapoxvirus (TANV) is one of the two identified members of the *Yatapoxviridae* genus, which are primate-specific poxviruses that infect monkeys and in humans cause mild monkeypox-like infections [97]. The Bcl-2-fold protein of tanapoxvirus, TANV16L (Figure 4d) [110] lacks recognizable sequence features such as the BH motifs [55], but nevertheless antagonizes cellular Bak and Bax in a yeast cell death assay [55]. Furthermore, biochemical interaction studies showed that TANV16L interacts with many pro-apoptotic Bcl-2 proteins with nanomolar to sub-micromolar affinities, with the exception of Noxa and Bok (Figure 2). Unusually, the crystal structure of TANV16L reveals both monomeric and domain-swapped dimeric configurations in two complexes that have been solved (Figure 4d–f). The TANV16L:BimBH3 and TANV16L:Bax BH3 complexes exist in a domain-swapped dimeric configuration [55], as previously seen for VACV F1L [56], VARV F1L [57], ORFV125 [59,108], and DPV022 [58]. In contrast to TANV16L:BimBH3, the TANV16L:PumaBH3 heterodimer complex exists as a monomeric complex, [55] as previously seen in M11L [51] and SPPV14 [102]. Analytical ultracentrifugation corroborated the structural data, showing that TANV16L exists as monomeric and dimeric forms, with a minor component of a heterotetrametric form. Site-directed mutagenesis of TANV16L binding groove residue R90 identified it as a key to interaction with pro-apoptotic Bcl-2 proteins, and the mutant R90A had a significantly reduced ability to interact with pro-apoptotic Bcl-2 proteins [55].

### 2.5. Herpesviridae

**Figure 5:**
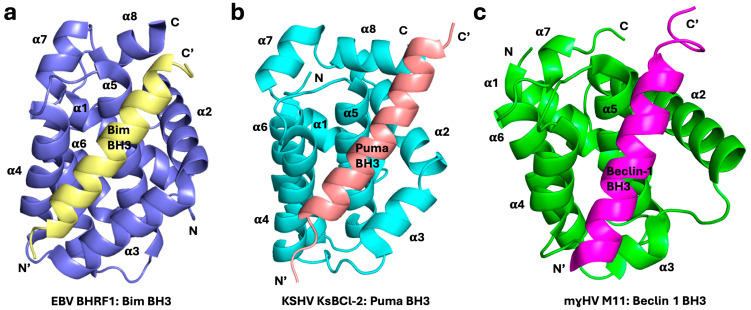
Structural comparison of herpesviral Bcl-2:BH3 peptide complexes. Cartoon diagrams of monomeric complexes in the Bcl-2 proteins. (**a**) Epstein–Barr virus BHRF1 (slate) in complex with Bim BH3 peptide (pale yellow) (PDB ID 2V6Q) [47]. (**b**) Kaposi sarcoma-associated herpesvirus KsBcl-2 (cyan) in complex with Puma BH3 peptide (salmon) (PDB ID 7QTX) [48]. (**c**) Murine gammaherpesvirus M11 (green) in complex with Beclin 1 BH3 peptide (magenta) (PDB ID 3DVU) [49]. The orientation of the cartoons is identical to that of Figure 3a.

Orthoherpesviruses are a member of the order *Herpesvirales*, which is made up of three families, the alpha, beta, and gamma herpesviruses, and are linear dsDNA viruses with genomes in the range of 125–295 kbp. Unlike poxviruses, they require the host cell nucleus for transcription, replication, and assembly. However, like the poxviruses, herpesviruses have acquired an array of genes that counteract the host’s apoptotic defenses and bear Bcl-2 homologs. These herpesviral Bcl-2 proteins share structural and functional similarities with cellular Bcl-2 family members (Figure 5) [15]. The herpesvirus-encoded Bcl-2 homologs are expressed during various stages of the viral life cycle and play pivotal roles in maintaining cell survival [15].

Epstein–Barr virus (EBV, Human herpes virus4, HHV4), a member of the γ-*Herpesviridae* family, is a widespread human herpesvirus. EBV infects human B-lymphocytes and transforms them into lymphoblastoid cells; it is associated with various diseases, including infectious mononucleosis and certain types of cancers, particularly lymphomas and nasopharyngeal carcinoma [111]. EBV infections have recently been correlated with multiple sclerosis [112]. EBV expresses two Bcl-2 homologs in its life cycle: BHRF1 and BALF1 [113]. BHRF1 is closely associated with mitochondrial membranes, plays a significant role in the viral life cycle, and contributes to the ability of EBV to evade host immune responses and promote oncogenesis [114]. BHRF1 is known for its anti-apoptotic function, akin to the cellular Bcl-2 family of proteins, and counteracts multiple host pro-apoptotic proteins, Bak, Bax, Bim, Bid, and Puma (Figure 2), to inhibit mitochondria-mediated cell death [46,47]. With its broad range of apoptotic ligands, BHRF1 blocks apoptosis induced by multiple apoptotic stimuli, such as etoposide, γ-irradiation, and cytosine arabinoside [47]. Furthermore, BHRF1 was shown to be expressed at considerably high levels in EBV-infected B-cells, thereby rendering them resistant to apoptosis. This anti-apoptotic activity is crucial for the persistence of EBV in the host [47]. Functionally, BHRF1 recruits Bim [115] and Bak [47] to interfere with intrinsic apoptosis. Structurally, BHRF1 adopts a monomeric Bcl-2 fold with eight α-helices (Figure 5a) [47,116]. While the helical fold of the canonical Bcl-2 binding groove is maintained in such vBcl-2 proteins, their sequence conservation is generally much lower than in their mammalian counterparts [7,13,15,117], thus often necessitating the determination of experimental structures. Site-directed mutagenesis identified F72 at the base of the binding groove, which is crucial for its interaction with Puma BH3 [118]. BHRF1 has been implicated in the development of EBV-associated cancers and resistance to treatment, particularly B-cell lymphomas [119]. As such, the expression of BHRF1 in hematopoietic stem and progenitor cells has been shown to upregulate MYC-induced lymphoma in mouse models [118].

The second EBV-encoded Bcl-2-like gene, BALF1, was initially identified as a pro-survival Bcl-2 protein [120], but a subsequent report presented an alternative view, indicating that BALF1 had pro-apoptotic properties and could inhibit BHRF1 [121]. Consequently, further research revealed that both BHRF1 and BALF1 play crucial roles in facilitating successful EBV-induced B-cell transformation [122]. Either BHRF1 or BALF1 were essential for the transformation of B-lymphocytes to lymphoblastoid cell lines and prevented newly infected cells from initiating apoptosis [122]. Knockout of both *balf1* and *bhrf1* genes led to B-cells infected with a double knockout virus undergoing apoptotic cell death [122]. Thus, both vBcl-2 genes in EBV play a role in the prevention of cellular apoptosis.

Kaposi’s sarcoma-associated herpesvirus (KSHV), an oncogenic γ-herpesvirus (human herpesvirus 8, HHV-8), encodes a viral Bcl-2 homolog, KsBcl-2, that is readily identifiable through the conservation of the BH1 sequence motif ‘NWGR’, frequently observed in Bcl-2 proteins [123]. KsBcl-2 is a key immunomodulatory protein expressed by KSHV and plays a significant role in the pathogenesis of Kaposi’s sarcoma, a cancerous tumor that primarily affects the skin and mucous membranes [124]. KsBcl-2 adopts the conserved Bcl-2 fold (Figure 5b) [48,125] and functions to inhibit the host intrinsic apoptosis pathway by interaction with multiple pro-apoptotic Bcl-2 proteins such as Bak, Bax, Bim, Bid, Bik, Bmf, Hrk, Noxa, and Puma [46,48] (Figure 2). The role of KsBcl-2 is primarily to enhance the survival of KSHV-infected endothelial cells [126]. Furthermore, KsBcl-2 was shown to be an essential component of the viral life cycle and the KsBcl-2 deletion mutant was unable to complete the viral life cycle [127,128]. Rhesus rhadinovirus, a γ-2 herpesvirus that infects rhesus macaque, encodes a vBcl-2 homolog, ORF16 [129], which was shown to functionally replace KsBcl-2 during the lytic cycle of viral replication [127]. However, the replacement of KsBcl-2 with cellular Bcl-x_L_ or previously characterized murine γ-herpesvirus Bcl-2 homolog M11 or the cytomegalovirus-encoded Bcl-2 homolog vMIA could not rescue KSHV replication [127], suggesting that KsBcl-2 may target additional host immunomodulatory functions.

Herpesvirus saimiri, an oncogenic herpes virus, also encodes putative vBcl-2 homolog ORF16, which bears the highly conserved BH1 and BH2 motifs. Functional data showed that ORF16 heterodimerized with mitochondrial apoptotic regulators Bak and Bak [130]. Also, ORF16 was shown to inhibit cell death in cell viability assays using Vero cells when cells were infected with recombinant Sindbis virus-encoding ORF16 [130].

Murine γ-herpesvirus 68 (mγHV68) is genetically and pathologically related to EBV and establishes persistent infection of B-cells. Like other gammaherpesvirus genomes, γHV68 contains a Bcl-2 homolog, M11 (or viral Bcl-2, vBcl-2) [131], and inhibits apoptosis induced by a number of stimuli, including viral infection [132]. The efficient infection of immature and transitional B-cells, but not other B-cell populations, requires the presence of M11 [132], and M11 mimics the function of the endogenous pro-survival Bcl-2 proteins [132]. Though M11 was initially identified as an extrinsic apoptotic inhibitor through its recruitment of Fas- and TNF-induced apoptosis [133,134], later biological, biochemical, and structural studies revealed that M11 is a Bcl-2 fold protein (Figure 5c) that interacts with pro-apoptotic Bcl-2 proteins Bak, Bax, Bim, Bid, Bmf, Hrk, Puma, and Noxa via its canonical ligand-binding groove [49].

Cytomegalovirus (CMV/human herpesvirus 5), a member of the β-*Herpesviridae* family, encodes the viral mitochondrial inhibitor of apoptosis (vMIA) that interacts with Bak [135] and Bax [136], inhibiting their oligomerization [136,137] and, thus, inhibiting mitochondrial-mediated apoptosis. vMIA does not share any sequence or structural features with Bcl-2 homologs and, surprisingly, the Bax–vMIA interaction does not involve the canonical ligand-binding groove on Bax [138] but, rather, the interaction site primarily employs the loops connecting α1–α2, α3–α4, and α5–α6 of Bax [137,138], where the vMIA Bax binding region forms a short helical segment [138]. Confoundingly, while human CMV neutralizes Bak and Bax, mouse CMV neutralizes Bak and Bax with two different proteins [139], CMV m38.5, which was shown to localize to mitochondria and target Bax [140,141], and m41.1, which targets Bak [142,143]. Together, these data suggest that vMIA plays a critical role in the CMV infection cycle and contributes to the ability of the virus to evade host immune responses.

Turkey herpes virus (HVT) is an α-herpesvirus that encodes a putative vBcl-2 homolog, vNR13/HVT079 [144], which shares sequence identity with cellular Bcl-B [145,146] (also known as Boo, Diva, or NRH in mammals and NRZ in zebrafish [147]), a poorly understood Bcl-2 homolog. These mammalian and zebrafish counterparts were shown to have a conserved Bcl-2 fold [7]. A recent study showed that vNR13/HVT079 localized to mitochondria and the endoplasmic reticulum [148]. In a functional study, vNR13/HVT079 expression inhibited apoptosis, a vNR13/HVT079 deletion mutant affected the viral replication in vitro, and the expression of vNR13/HVT079 disrupted mitochondrial morphology in HVT-infected cells [148]. These data indicate that vNR13 functions by inhibiting apoptosis [148].

## 3. Virus-Encoded Bcl-2 Proteins Modulate Autophagy

Like their response to apoptotic cell death, viruses have adapted to exploit autophagy, either to activate it or inhibit it for successful infection and replication. The ability of cellular Bcl-2 proteins to mediate autophagy is well established [149,150] and, similarly, certain virus-encoded Bcl-2 proteins are involved in autophagy regulation. Autophagic cell death represents another form of RCD in addition to apoptosis, and both mechanisms of RCD are likely in virally infected cells [1]. vBcl-2 proteins have been implicated in mediating autophagy and, mechanistically, there are close similarities with cellular Bcl-2 proteins and the BH3-in-groove binding mode as well as a close association with intracellular membranes. The ASFV A179L protein binds the BH3-like motif of Beclin 1 (Figure 3), a key protein in the assembly of autophagosomes, and inhibits autophagosome assembly in Vero cells [63]. In cell-based assays, A179L was able to prevent autophagosome formation after starvation [151]; however, the direct impact of autophagy on the outcome of ASFV infection and viral titers remains to be clarified.

Autophagy signaling via Beclin 1 is emerging as a principal target for certain *Herpesviridae*. In particular, EBV BHRF1 downregulates Beclin 1 starvation-induced autophagy in human breast adenocarcinoma cells, and it has been demonstrated in at least two hybrid experiments that BHRF1 interacts directly with Beclin 1 [152]. Immunoprecipitation experiments performed in HeLa cells also demonstrated that BHRF1 was associated with Beclin 1 in transiently transfected cells and promotes the fragmentation and sequestration of mitochondria in autophagosomes [153]. This process leads to mitochondrial degradation through mitophagy, the autophagic destruction of mitochondria. While there is less data available for BALF1, the second Bcl-2 protein of EBV, it co-localizes with autophagosomes upregulating autophagy [154]. KsBcl-2 from KSHV was also shown to interact with Beclin 1, downregulate autophagy, and modulate autophagosome formation in the host cell [155]. The interference of autophagy through vBcl-2 represents a common theme for KSHV and EBV, indicating the importance of regulating autophagy to the virus.

The most compelling evidence for a major role of autophagy during herpesvirus infection comes from studies on murine γ-HV68 and its encoded Bcl-2 homolog M11 [131]. M11 inhibited host autophagy signaling by interacting with the BH3 motif of Beclin 1 [155], similar to what was observed with key pro-apoptotic Bcl-2 proteins. Biochemical and structural investigations have shown the molecular determinates of Beclin 1-BH3 bind to M11 [156]. The M11–Beclin 1 interaction is much tighter (nM vs mM K_d_) when compared to that observed with KsBcl-2 or cellular Bcl-2 [155], suggesting that M11 may primarily target the inhibition of autophagy rather than apoptosis. E et al. showed that the anti-autophagy and anti-apoptosis activities of vBcl-2 are functionally distinct [157]. vBcl-2:Beclin 1 interference with autophagy was necessary for latent infection, while the anti-apoptotic function was necessary for viral reactivation after latency.

Beyond ASFV and herpesviruses, other viruses have developed the ability to interfere with autophagy, though, frequently, the molecular basis of autophagy activation remains to be elucidated. A common theme is the ability of viral proteins to interact with Beclin 1 to disrupt autophagosomes at various stages through binding either Bcl-2 homologs or other viral proteins, mimicking the ability of cellular Bcl-2 [149]. Autophagy enhancement, rather than its disruption, is also a strategy employed by viruses. Adenovirus infection, rather than disrupting autophagy, induces autophagy when the adenovirus Bcl-2 homolog, E1B19K, interacts with Beclin 1 [158]. Autophagosomes were directly observed when the captipoxvirus Lumpy Skin Disease Virus (LSDV) infected bovine embryonic fibroblasts, suggesting that autophagy is directly involved in mediating cell death [159]. While autophagy may be initiated to destroy viruses through lysosomal degradation (xenophagy) and destroy gene products (virophagy) or organelles (mitophagy) to prevent apoptosis as part of an innate immunity response, it appears that multiple different levels of interference are employed by dsDNA viruses [160]. Autophagy is a coordinated process that has multiple protein components in mammalian cells [33], and this complexity gives viruses a wide scope to access many points of control. The focal point of the viral Bcl-2 homologs appears to be Beclin 1 binding and autophagosome regulation.

## 4. Specificities and Affinities of BH3 Peptide Binding by Viral and Mammalian Bcl-2 Proteins

There are a wide range of BH3 ligand specificities for the viral Bcl-2 homologs, but most bind Bak, Bax, Bid, Bim, and Puma, the most potent pro-apoptotic proteins, with moderate to high affinity (Figure 2). Beclin 1 is only bound by selected viral Bcl-2 proteins, including the herpesviruses vBcl-2 KSHV, KsBcl-2, and M11 and the asfarvirus ASFV A179L. Strikingly, the grouper iridovirus Bcl-2 GIV66 is highly specific, binding to Bim only. These specificities likely reflect the importance of the particular apoptotic pathways selected for the cell type infected. Furthermore, the distinct interaction profiles also speak to the defined infection sites and targets for each virus, and reflect the degree of specialization of each pathogen for its host.

## 5. Conclusions

Poxviruses and iridoviruses have been associated with sponges, though cellular infection has not been demonstrated yet [161]; thus, from an evolutionary perspective, the accumulation of Bcl-2 genes and their preservation in dsDNA viral genomes is not surprising. Apoptosis and autophagy are both highly regulated, evolutionarily conserved processes, not only essential for homeostasis, development, and innate immunity but also targets for viral manipulation. It is likely that viral manipulation of both apoptosis and autophagy is required for cell survival during viral invasion, and the Bcl-2 family and mimics play a central role in this process. Viral Bcl-2 proteins have a high degree of structural homology (as demonstrated in Figure 2, Figure 3 and Figure 4), though not always a high degree of shared sequence identity with their metazoan or viral counterparts. In addition, while some viral Bcl-2 proteins share a high degree of sequence identity with other poxviral Bcl-2 proteins, they do not necessarily share an exact functional identity.

Viral Bcl-2 proteins interact with BH3 ligands of pro-apoptotic Bcl-2 proteins and Beclin 1 through the canonical BH3-in-groove interaction to effect their action on apoptosis and autophagy. Structural studies have demonstrated that the intermolecular interactions between the BH3 ligand and the Bcl-2 protein are highly homologous. Multiple biochemical and structural studies have confirmed that vBcl-2 proteins interact with pro-apoptotic cellular Bcl-2 proteins including Bax, Bak, and the BH3-only group [15]. Structural studies have confirmed the BH3-in-groove mode of interaction between the pro-survival vBcl-2 and BH3 motifs of cellular pro-apoptotic proteins and with the BH3-like motif of Beclin 1 (Figure 3) [15]. However, novel modes of interaction have also been observed between viral proteins and the mammalian Bcl-2 family, as demonstrated with vMIA, which binds pro-apoptotic Bax in a non-BH3-binding groove mode [138].

Understanding the molecular basis of the mechanism of action of viral Bcl-2 proteins and their targets may shed light on their importance in host–virus adaptation and how they avoid or minimize the host immune response. Promising results from the genetic deletion of the Bcl-2 homolog, A179L, in ASFV [41] show that manipulating the viral regulation of apoptosis provides a possible route to control viral replication by antagonizing pro-survival Bcl-2 proteins. Other modes of action have been hypothesized for viral Bcl-2 proteins; a recent proteomics screen indicated that EBV BHRF1 interacts with the pattern recognition receptor and that mitochondrial and associated membranes localized Mitochondrial Anti-Viral Signalling (MAVS) protein [162], a protein required for the innate immune antivirus response. The ability of viruses to manipulate cellular death induction through apoptosis and autophagy by the expression of viral pro-survival Bcl-2 proteins provides the prospect of targeting this family in viral disease. The direct targeting of Bcl-2 proteins has proved a productive strategy for treating diseases that are dependent on the Bcl-2 family for cell survival [163].

## Data Availability

Not applicable.
